# Silver Nanowires as Electron Transfer Mediators in Electrochemical Catechol Biosensors

**DOI:** 10.3390/s21030899

**Published:** 2021-01-29

**Authors:** Coral Salvo-Comino, Fernando Martin-Pedrosa, Cristina Garcia-Cabezon, Maria Luz Rodriguez-Mendez

**Affiliations:** 1 Group UVASENS, Escuela de Ingenierías Industriales, Universidad de Valladolid, Paseo del Cauce, 59, 47011 Valladolid, Spain; coraldeugena@hotmail.com; 2 BioecoUVA Research Institute, Universidad de Valladolid, 47011 Valladolid, Spain; fmp@eii.uva.es; 3 Department of Materials Science, Universidad de Valladolid, Paseo del Cauce, 59, 47011 Valladolid, Spain

**Keywords:** silver nanowires, tyrosinase, electrochemical biosensor, catechol

## Abstract

The integration of nanomaterials as electron mediators in electrochemical biosensors is taking on an essential role. Due to their high surface-to-volume ratio and high conductivity, metallic nanowires are an interesting option. In this paper, silver nanowires (AgNWs) were exploited to design a novel catechol electrochemical biosensor, and the benefits of increasing the aspect ratio of the electron mediator (nanowires vs. nanoparticles) were analyzed. Atomic force microscopy (AFM) studies have shown a homogeneous distribution of the enzyme along the silver nanowires, maximizing the contact surface. The large contact area promotes electron transfer between the enzyme and the electrode surface, resulting in a Limit of Detection (LOD) of 2.7 × 10^−6^ M for tyrosinase immobilized onto AgNWs (AgNWs-Tyr), which is one order of magnitude lower than the LOD of 3.2 × 10^−5^ M) obtained using tyrosinase immobilized onto silver nanoparticles (AgNPs-Tyr). The calculated K_M_ constant was 122 mM. The simultaneous use of electrochemistry and AFM has demonstrated a limited electrochemical fouling that facilitates stable and reproducible detection. Finally, the biosensor showed excellent anti-interference characteristics toward the main phenols present in wines including vanillin, pyrogallol, quercetin and catechin. The biosensor was able to successfully detect the presence of catechol in real wine samples. These results make AgNWs promising elements in nanowired biosensors for the sensitive, stable and rapid voltammetric detection of phenols in real applications.

## 1. Introduction

Phenolic compounds are relevant in the food industry due to their antioxidant properties and health benefits. They are also responsible for some important organoleptic characteristics of foods and affect their antioxidant properties [1]. Flavonoids are an important class of phenolic compounds contained in both wines and grapes. Anthocyanins and flavanols are the two main groups of flavonoids present in red wines. They have a strong influence on the organoleptic properties of wines such as color, astringency, and bitterness. In addition, they are very active chemically and are involved in several chemical reactions during winemaking and aging. The assessment of certain polyphenols (such as catechol and catechol derivatives) is crucial to improve the quality of wines. The assessment of phenolic compounds in wines is usually carried out using chromatographic or spectroscopic techniques [2]. Electrochemical biosensors are an alternative, due to their selectivity, portability, and low cost. They can overcome the limitations of traditional methods [3], and their performance can be improved using nanomaterials as electron mediators [4].

In the last few years, a number of electrochemical biosensors combining phenol oxidases with a variety of materials, such as polymeric matrices [5,6] or nanomaterials, have been developed [7,8]. Carbon nanotubes (CNTs) [9,10] graphene [11,12], carbon fibers [13], metallic nanoparticles, metal oxide nanoparticles, nanostars [14,15,16,17,18], molecular imprinted polymers [19], and nanostructured thin films [20,21] have all been shown to be excellent electron mediators. One-dimensional (1D) nanostructures can be an interesting alternative to zero-dimensional (OD) nanoparticles due to their high surface-to-volume ratios. Only a few studies have explored the advantages of nanowires (mainly carbon-based) as electron mediators [22].

During the last few years, silver nanowires have attracted interest due to their high conductivity and excellent thermal properties. The applications include transparent conductors, adhesive materials, electro-actuators, electronic devices, etc. [23,24,25]. They have also been used as sensing material in nonenzymatic electrochemical sensors [26,27,28,29]. In spite of this interest, only four examples of AgNWs have been reported in biosensor applications. These include three reports of glucose biosensors [30,31,32] and one of a cholesterol biosensor [33]. Until now, phenol oxidases have not been immobilized on AgNWs.

These previously published works show promising results, though many aspects need to be analyzed further. First, the immobilization capabilities and electron mediator properties must be analyzed in detail and compared with their nanoparticle counterparts. Another aspect that needs to be improved is control of the AgNWs size. AgNWs used in previous works were prepared by different methods, including the polyol process. However, whatever the method used, the AgNWs obtained are inhomogeneous in size [34,35,36,37]. Controlling the size of AgNWs could be a useful strategy to obtain improved and reproducible devices.

Finally, the use of atomic force microscopy (AFM) techniques can provide information about the structure of nanowires and the quality of the enzymatic coverage. Moreover, the simultaneous use of cyclic voltammetry and AFM (CV_AFM) is emerging as a powerful tool to analyze the surface changes occurring during the electrochemical processes. This information can be used to understand the fouling processes that usually limit the lifetime of biosensors [38].

In this work, a novel electrochemical biosensor was developed for the detection of catechol, based on tyrosinase immobilized onto AgNWs (AgNWs-Tyr). The sensitivity and detection limit of the developed biosensor was evaluated by voltammetric techniques.

During the work, several aspects were analyzed and/or optimized. For instance, the synthesis of AgNWs following the polyol method was optimized to obtain homogeneous nanowires. The adequate immobilization of the enzyme on the AgNWs was controlled by AFM. The simultaneous use of cyclic voltammetry and AFM (CV_AFM) was used to analyze fouling processes. Finally, the capability of the developed biosensor to detect catechol in the presence of other phenols typically found in foods (including vanillin, pyrogallol, quercetin, or catechin), was analyzed.

## 2. Materials and Methods

### 2.1. Chemicals

Silver nitrate (AgNO_3_), polyvinylpyrrolidone (PVP, Mw = 55,000), ethylene glycol (EG) anhydrous (99.8%), tyrosinase (Tyr from *Agaricusbisporus* activity of 1000 U mg^−1^, CAS 9002-10-2), phosphate-buffered saline (PBS), ethanol, catechol, vanillin, pyrogallol, quercetin, and catechin were purchased from Sigma-Aldrich (Saint Louis, MO, USA). Glutaraldehyde (50% aqueous solution) was purchased from Alfa Aesar (Haverhill, MA, USA). Deionized water from MilliQ (Millipore-Sigma Aldrich, Darmstadt, Germany) (resistivity 18.2 MΩ·cm) was used in all experiments. Indium tin oxide (ITO) glass substrates were purchased from Sigma-Aldrich (Darmstadt, Germany).

### 2.2. Instruments

A Perimax peristaltic pump from Spetec and a Sorvall ST 8 Centrifuge (Thermo Scientific, Walthman, MA, USA) were used for the synthesis of the AgNWs. Films were prepared using a Spincoater 1H-D7 device (Mikasa, Tokyo, Japan). UV–Vis characterization of AgNWs dispersions was performed using a UV-2600 device (Shimadzu., Duisburg, Germany) Fourier Transform Infrared (FTIR) spectra of films deposited on ZnS substrate were obtained using an FTIR 6600 spectrophotometer (Jasco, Pfungstadt, Germany) from 700 to 4000 cm^−1^, at a resolution of 4 cm^−1^ and 1000 scans.

X-ray diffraction (DRX) was performed using a Bruker Discover D8 (Bruker, Ettlingen, Germany) with a wavelength of 1.5418 Å. Transmission electron microscopy (TEM) images were obtained with a JEM 1011HR microscope (Jeol, Freising, Germany). Atomic force microscopy (AFM) images were recorded in films deposited on ITO at room temperature in a Cypher ES Environmental AFM device (Oxford Instruments, Asylum research, Wiesbaden, Germany) operated in tapping mode with blueDrive photothermal excitation technology. The tip used was an AC160TSA-R3 (Oxford Instruments, Asylum research, Wiesbaden, Germany). Electrochemical measurements were carried out using a PGSTAT128 potentiostat/galvanostat (AutolabMetrohm, Utrecht, The Netherlands).

### 2.3. Preparation of AgNWs

AgNWs were synthesized following a modification of the polyol method, consisting in the reduction of a silver precursor (AgNO_3_) in the presence of EG as the reducing agent. PVP was used as a surfactant to facilitate the AgNW dispersion and CuCl_2_ was added to promote the growth of AgNWs and to prevent the presence of free Ag^+^ ions in the solution through the formation of AgCl products [34].

Even though the polyol process is an efficient method, AgNWs are typically synthesized within a certain range of lengths and diameters, depending on various factors such as the reaction time, temperature, velocity of addition of the metallic precursor and the surfactant, and use of a mediated agent.

In this work, the process was optimized to obtain homogeneous AgNWs. In detail, 0.5 mL of CuCl_2_ (1.5 × 10^−4^ M, in EG) was added to 5 mL of EG previously heated in a round bottom flask and maintained under stirring (200 rpm) and reflux (the temperature was kept constant at 160 °C during the procedure). After 5 min, 5 mL of PVP (0.36 mM in EG) and 2.5 mL of AgNO_3_ (0.12 M in EG) were pumped dropwise simultaneously over 10 min (special attention to the pump speed of each component must be paid in order to obtain homogeneous nanowires: flow rates were 0.25 mL/min for AgNO_3_ and 0.5 mL/min for PVP). The reaction was maintained for 1 h until the silver precursor was completely reduced. The AgNWs were separated from the Ag nanoparticles that might have appeared by centrifugation (2000 rpm for 20 min). Seven washing cycles with ethanol were carried out until the yellow color of the supernatant (corresponding to the presence of AgNPs) was no longer observed (these AgNPs were used in the experiments carried out for comparison purposes). Finally, the purified AgNWs were resuspended in ethanol and kept in darkness at room temperature.

### 2.4. Preparation of AgNWs-Tyr Modified Electrode

AgNWs were deposited onto ITO substrates by adding 50 μL AgNWs suspension (2 g/L in ethanol) using the spin coating method (120 s at 1000 rpm). Then, 50 μL of Tyr (5 g/L in PBS 0.01 M, pH 7.0) was drop-casted onto the electrode surface. The electrode was then exposed to glutaraldehyde vapors (25% aqueous solution) for 10 min. After drying overnight at room temperature, the biosensor was washed with a PBS 0.01 M solution.

## 3. Results and Discussion

### 3.1. Characterization of AgNWs

**Figure 1:**
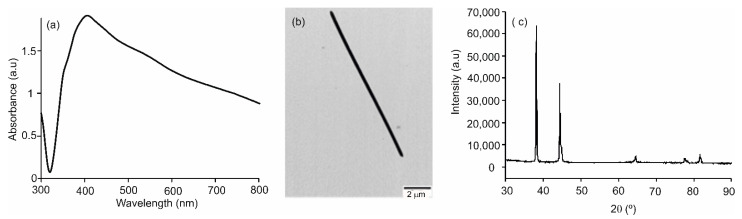
(**a**) UV–Vis absorption spectrum of the ethanol suspension; (**b**) TEM image and (**c**) XRD pattern of the purified silver nanowires (AgNWs).

AgNWs were prepared using a modified polyol method that allowed us to obtain nanowires with homogeneous sizes. Figure 1a shows the UV–Vis in an absorption spectrum of the purified AgNWs resuspended in ethanol. The spectrum showed a peak at 402 nm, accompanied by a shoulder at 355 nm, which are considered the AgNWs fingerprint [39]. TEM images (Figure 1b) revealed homogeneous metallic nanostructures with an average diameter of 400 ± 20 nm and 10 ± 0.05 µm in length. The XRD pattern is shown in Figure 1c. As Ag arranges in the fcc structure, the five sharp peaks, displayed at 2θ angles of 38°, 44°, 64°, 77° and 82°, correspond to the [111], [200], [222], [311] and [400] crystalline planes. The high intensity of the first two peaks suggested that the AgNWs were preferably oriented along the [111] and [200] planes [31].

**Figure 2:**
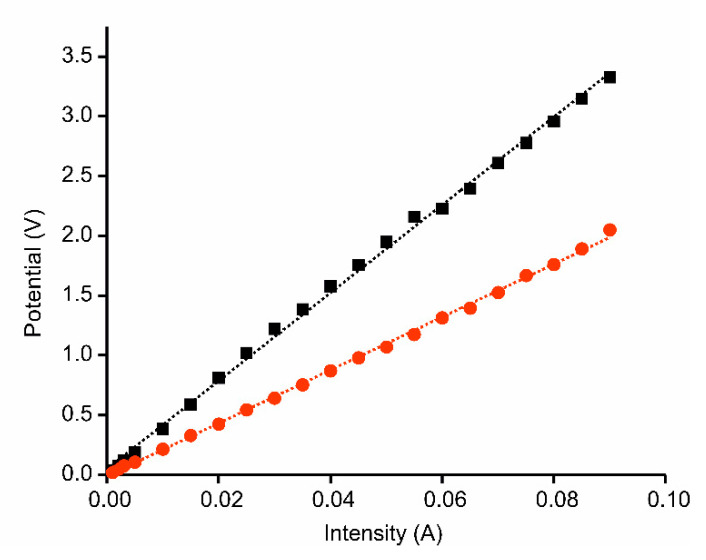
Intensity vs. potential regression curves of indium tin oxide (ITO) (black) and ITO-AgNWs (red).

The resistivity of a thin film of AgNWs (deposited on ITO by means of spin coating), was tested by means of the four point probe test. The resistivity (*ρ*) was calculated using Equation (1):

(1) $$\rho =\frac{\pi }{\ln\left(2\right)}\left(\frac{V}{I}\right)t=4.532\left(\frac{V}{I}\right)t$$

where *V* is the voltage measured between the inner probes, *I* is the intensity flowing through the circuit (between the outer probes), and *t* is the film thickness (which is equal to 1 if the thickness is negligible). Results are shown in Figure 2. As expected, the presence of AgNWs increased the conductivity of the electrode. This effect could be advantageous in sensing applications.

### 3.2. Characterization of the AgNWs-Tyr Biosensors: Spectroscopy and AFM

**Figure 3:**
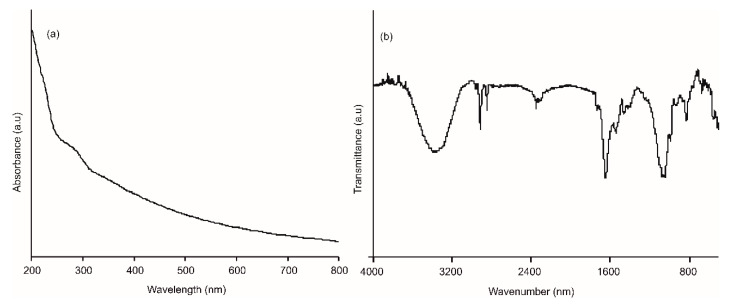
(**a**) UV–Vis absorption spectrum and (**b**) FTIR spectrum of the AgNWs-Tyr biosensor.

A buffer solution of the enzyme was drop-casted onto the AgNWs film to obtain the AgNWs-Tyr biosensor. UV–Vis, FTIR and AFM analysis were performed to characterize the structure of the biosensor and confirm the immobilization of the enzyme. The UV–Vis spectrum of the biosensor clearly shows the features corresponding to the AgNWs, along with a characteristic absorption peak at 280 nm due to the presence of the amino acid tyrosine (Figure 3a). The adsorption of tyrosinase was corroborated by the FTIR spectrum (Figure 3b), where one band at 1100 cm^−1^ associated to the presence of hydroxyl groups, and two characteristic bands at 1550 and 1650 cm^−1^ (corresponding to the presence of the enzymatic Amide II and I, respectively) could be observed [40].

**Figure 4:**
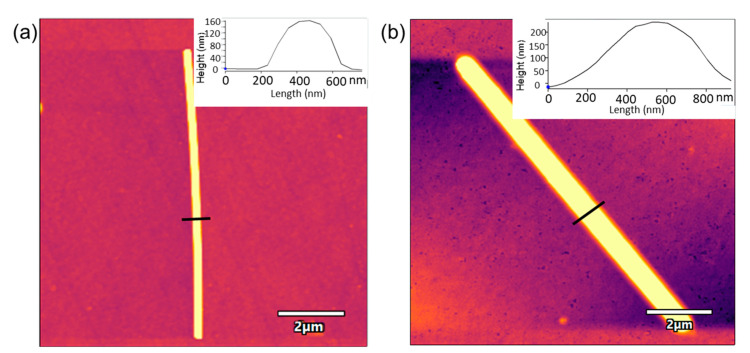
AFM images of (**a**) ITO-AgNWs sensor, (**b**) AgNWs-Tyr biosensor. Insets correspond to the line profile analysis.

AgNWs were also characterized using AFM. As observed in Figure 4, AgNWs deposited onto the ITO glass showed a length of ~10 µm and a width of ~400 nm. The AFM image of Tyr deposited on the ITO-AgNWs surface showed a highly homogeneous distribution of the enzyme on the surface of the metallic nanowires. The width of the AgNWs-Tyr nanostructure increased uniformly (width ~600 nm). This can be explained by the high affinity between AgNWs and tyrosinase, which maximized the contact surface, reducing the insulating effects while facilitating the electron transfer—as demonstrated in the next section.

### 3.3. Electrochemical Characterization of the AgNWs-Tyr Biosensor

**Figure 5:**
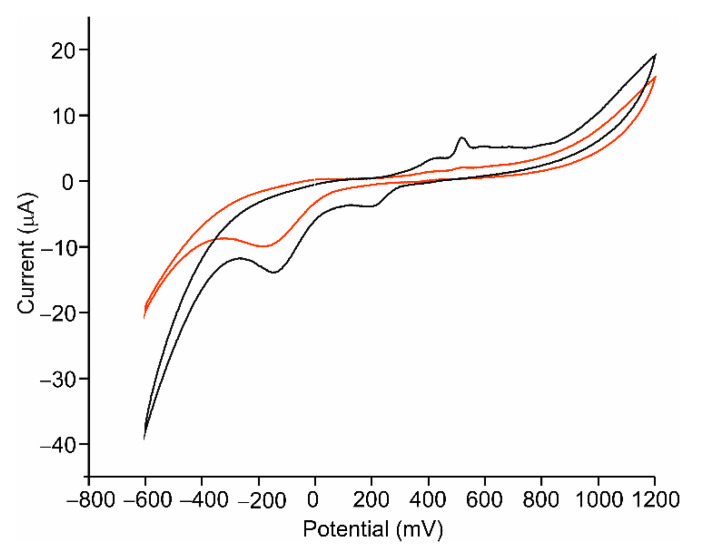
Voltammetric response in catechol 10^−4^ mol·L^−1^ in 0.01 mol·L^−1^ phosphate-buffered saline (PBS), pH 7.0 of a bare ITO (red) and ITO-AgNWs (black).

Cyclic voltammetry (CV) was used to study the electrochemical response of the AgNWs-Tyr biosensor. Experiments were carried out in catechol 10^−4^ M in PBS solution 0.01 M, pH 7.0, at a scan rate of 100 mV/s. The responses of bare ITO and ITO covered by AgNWs to catechol were registered for comparison purposes and are shown in Figure 5. The response of ITO to catechol was characterized by a cathodic peak at −150 mV, produced by the reduction of the o-quinone (obtained by the oxidation of catechol [17,40,41,42]. The intensity of the peak increased when the ITO was covered with AgNWs (−13 μA for bare ITO and −20 μA for ITO-AgNWs). In addition, a redox pair corresponding to the Ag^+^/Ag process was also observed at 268 mV (cathodic wave) and 595 mV (anodic wave).

**Figure 6:**
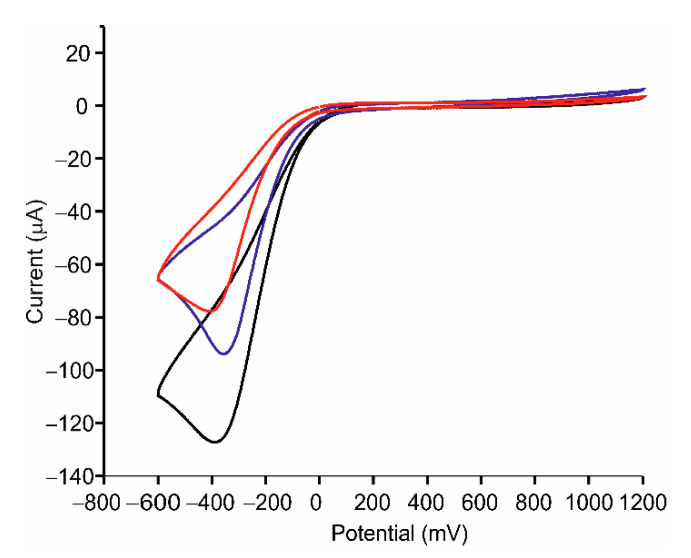
Voltammetric response in catechol 10^−4^ mol·L^−1^ in 0.01 mol·L^−1^ PBS, pH 7.0 of ITO-Tyr (red), AgNPs-Tyr (blue) and AgNWs-Tyr (black).

When Tyr was deposited onto the ITO electrode, the intensity of the peak corresponding to the reduction of o-quinone was clearly higher than in the absence of the enzyme (−13 μA in bare ITO and −77 µA for ITO-Tyr). The intensity of the cathodic peak increased in the presence of spherical silver nanoparticles (−93 µA for AgNPs-Tyr). This intensity increased even further in the presence of nanowires (−128 µA for AgNWs-Tyr) (Figure 6). This result evidenced the influence of the shape of the silver nanomaterial and confirmed that AgNWs, having a higher aspect ratio than AgNPs, improved the electron transfer between the enzyme and the electrode.

**Figure 7:**
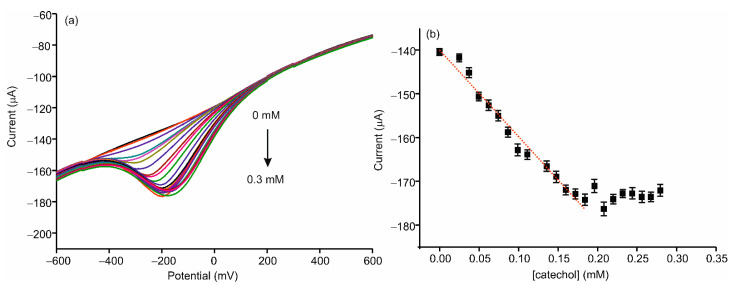
(**a**) Differential pulse voltammetry (DPV) response in 0.01 mol·L^−1^ PBS, pH 7.0 to an increase concentration of catechol of an ITO-AgNWs–Tyr, (**b**) calibration curve of the response for increasing concentration of catechol.

Differential pulse voltammetry (DPV) was used to evaluate the response of the AgNWs-Tyr biosensor to increasing concentrations of catechol in PBS solution 0.01 M, pH 7.0, at a scan rate of 100 mV/s. As observed in Figure 7, the current intensity increased linearly with the catechol concentration from 24.9 to 172 µM, following the equation y = (−197.9 ± 8.61) × (−139.98 ± 0.95)(R^2^ = 0.9759). The limit of detection (LOD), calculated following the 3·σ/m criterion, was 2.7·10^−6^ M for AgNWs-Tyr, whereas the LOD obtained using an AgNPs-Tyr biosensor (prepared under the same conditions) was 3.2 × 10^−5^ M. In addition, the sensitivity calculated for the AgNW-Tyr biosensor was 197.9 µA·mM^−1^ which is one to two orders of magnitude higher than the sensitivity shown by other sensors combining tyrosinase and silver nanoparticles reported in recent papers [16,43]. Some results obtained with biosensors containing AgNPs or AuNPs are collected in Table 1.

Finally, the apparent Michaelis–Menten ($K_{M}^{app}$) constant was calculated following the Lineweaver–Burk approach by representing 1/I_lim_ vs. 1/[S_ox_], where I_lim_ is the intensity of the maximum and Sox is the substrate concentration (catechol in this case) [46]. These results confirm the excellent performance of AgNWs as electron mediators, probably due to the excellent affinity between the AgNWs and the enzyme.

**Figure 8:**
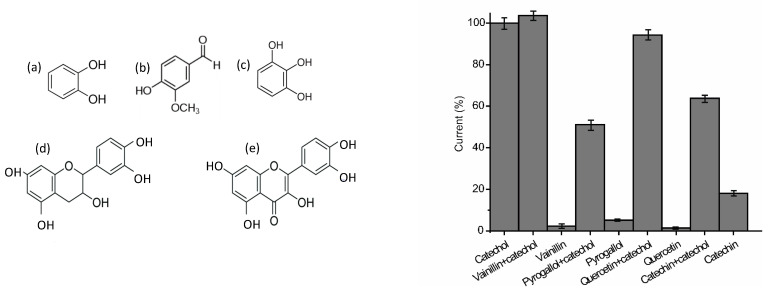
**Left:** Phenols included in the study, (**a**) catechol, (**b**) vainillin, (**c**) pyrogallol, (**d**) catechin and (**e**) quercetin. **Right:** Cathodic maximum peak intensity obtained immersing the AgNWs-Tyr sensor in 10^−4^ M catechol (in 0.01 mol·L^−1^ PBS pH 7.0) in the presence of interfering phenols.

The selectivity of the AgNWs-Tyr biosensor was evaluated by analyzing the response to catechol (a diphenol) in the presence of other phenols usually found in foods including vanillin (a monophenol) and pyrogallol (a triphenol). Two flavonoids containing a resorcinol moiety linked to a catechol moiety (quercitin and catechin) were also included in the study. Experiments were carried out using a 2 × 10^−4^ M solution of catechol that was diluted 1:1 (*v*:*v*) with a 2 × 10^−4^ M solution of the corresponding interferent. The intensities of the cathodic peaks at ~−400 mV were compared with the response of 10^−4^ M solutions of the pure components. As shown in Figure 8, the AgNWs-Tyr biosensor did not respond to other phenols usually present in foods, confirming the high degree of selectivity toward this phenol. This result can be explained taking into account that tyrosinase has high affinity toward o-diphenols, whereas it has a limited affinity toward triphenols and polyphenols. This justifies the small response observed toward solutions of vanillic acid, pyrogallol, quercitin or catechin. The decrease in the responses toward mixtures of catechol:pyrogallol, catechol:quercitin, or catechol:catechin can be explained by assuming that pyrogallol, catechin and quercitine can block some active sites, reducing the response of the biosensor to catechol.

### 3.4. In Situ EC-AFM Studies. Reproducibility and Repeatability

In recent years, the simultaneous use of electrochemistry and AFM has emerged as a useful technique to study the electrode surfaces and surface phenomena occurring during the electrochemical process. The repeatability of the reported AgNWs-Tyr biosensors was evaluated by measuring 15 consecutive cycles in catechol 10^−4^ M in PBS solution 0.01 M, pH 7.0, at a scan rate of 100 mV/s from −600 to 1200 mV. The calculated variation coefficient was 3.2%, demonstrating the effective immobilization of the enzyme with a minimal loss of enzymatic activity and minimal fouling upon successive cycling.

**Figure 9:**
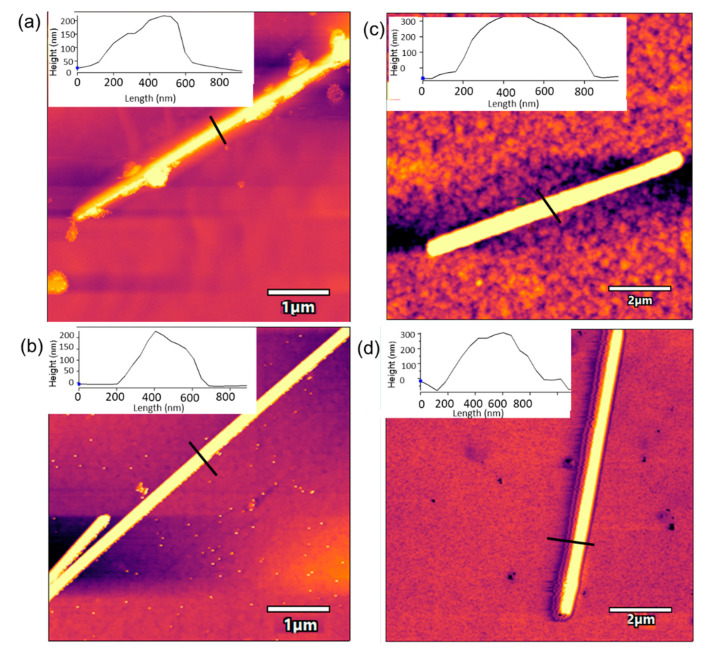
AFM image of (**a**) ITO-AgNWs sensor after voltammetry, (**b**) ITO-AgNWs sensor after voltammetry and washing with water, (**c**) AgNWs-Tyr biosensor after voltammetry and (**d**) AgNWs-Tyr biosensor after voltammetry and washing with water.

Surface changes occurring during cycling were further analyzed by AFM. Figure 9 shows the topography of ITO-AgNWs (left) and ITO-AgNWs-Tyr (right) before and after voltammetry.

AFM images registered after 15 consecutive cycles demonstrated the formation of precipitates that were mainly concentrated on the surface of the metallic structure. In the case of the ITO-AgNWs surface, precipitates were large and homogeneously distributed along the AgNWs surface, whereas in the AgNWs-Tyr biosensor, the precipitates uniformly covered the surface of the AgNWs, filling the holes and spaces left by the enzyme during its deposition. These precipitates could easily be removed by washing with water, indicating that the precipitates were produced by crystallites of the saline buffer, or by polyphenol weakly bound to the surface. Moreover, after washing, the original size was restored and the initial voltammograms could be reproduced with only minor differences with respect to the original one. According to these results, it can be concluded that the presence of AgNWs can prevent the nonspecific adsorption of fouling agents.

**Figure 10:**
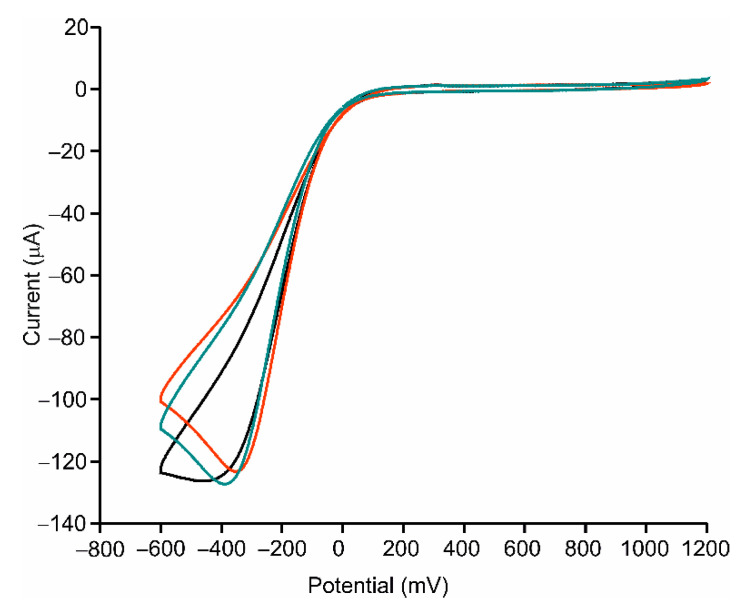
Voltammetric response in catechol 10^−4^ mol·L^−1^ in 0.01 mol·L^−1^ PBS, pH 7.0 of three different AgNWs-NoTyr biosensors prepared under the same condition.

Finally, in order to evaluate the reproducibility of the AgNWs-based device, the voltammetric responses of three devices, prepared under the same conditions, were observed; these showed a relative standard deviation (RDS) of 1.65%. The excellent reproducibility could be attributed, at least in part, to the size homogeneity of the AgNWs obtained using our improved synthetic method that can help to minimize differences from one device to another (Figure 10).

**Figure 11:**
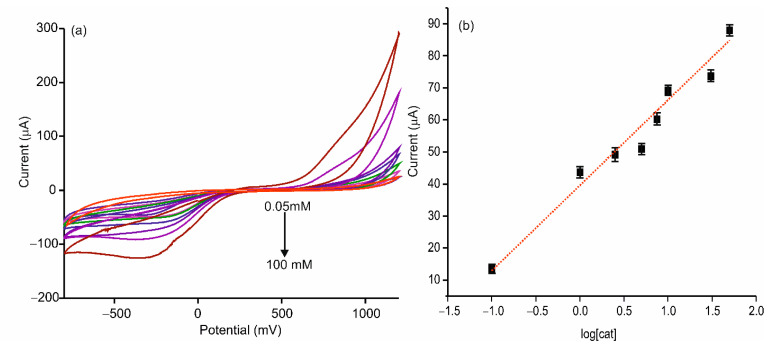
(**a**) Voltammetric response in a diluted wine 10% in PBS 0.01 M to addition of increasing concentrations of catechol (5 × 10^−5^, 10^−4^, 2.5 × 10^−4^, 5 × 10^−4^, 7.5 × 10^−4^, 10^−3^, 5 × 10^−3^, 10^−2^ M) in 0.01 mol·L^−1^ PBS, pH 7.0, (**b**) calibration curve of the response for increasing concentration of catechol.

Finally, the developed nanowired biosensor AgNWs-Tyr was used to assess the catechol concentration in a commercial red wine using the standard addition method (addition of 10^−5^, 5 × 10^−5^, 10^−4^, 2.5 × 10^−4^, 5 × 10^−4^, 7.5 × 10^−4^, 10^−3^, 5 × 10^−3^, 10^−2^ M catechol in wine diluted 10% in PBS 0.01 M). The values obtained corresponded to the mean of three determinations and their subsequent associated standard deviations. The obtained voltammograms and the calibration curve are represented in Figure 11, [y = (26.64 ± 2.09)·log(x) + (39.56 ± 1.08)] (R^2^ = 95.8115). The calculated concentration of catechol in the diluted wine sample was 32 µM, which is in good agreement with the results obtained in previous works based on electrochemical sensors for catechol detection in red wines [19].

## 4. Conclusions

Silver nanowires were used as a platform for the immobilization of tyrosinase, leading to a catechol biosensor with excellent performance. The nanowires showed a high affinity toward the enzyme, and the high aspect ratio facilitated the electron transfer. The AgNWs-Tyr biosensor presented a linear range from 24.9 to 172 µM, and a detection limit of 2.7 × 10^−6^ M. It showed a high sensitivity and low KM_app_ values, related to the good accessibility of catechol molecules to the active site of the enzyme. The excellent reproducibility and repeatability obtained could be attributed to the homogeneity of the AgNWs and their ability to prevent fouling effects. The implementation of the biosensor in a wine complex matrix demonstrated the feasibility of incorporating the developed biosensor in field applications.

## Figures and Tables

**Table 1:** Sensing parameters of different phenol oxidases modified electrodes toward catechol.

Biosensor Description	R^2^	Sensitivity (µA mM^−1^)	LOD (M)	Linear Range (µM)	Ref.
AgNWs-Tyr	0.976	197.9	2.7·10^−6^	25–172	This work
Lac/AgNPs/cellulose/GCE	0.999	11.5	1.64·10^−6^	5–3650	[43]
Gr/PPy/AgNPs/PPO	--	13.66	4.70·10^−7^	1–15	[16]
Tyr-AuNPs-SPCE	0.993	0.55	1.2·10^−6^	2.5–20	[15]
Tyr-AuNPs-DHP/GCE	0.999	115	1.7·10^−5^	2.5–95	[44]
PEDOT/PSS/AuNPs-Tyr	0.971	--	2.8·10^−6^	90–1500	[45]

Gr: graphene, PPy: polypyrrol, AgNPs: silver nanoparticles, PPO: polyphenol oxidase, GCE: glassy carbon electrode, DHP: dihexadecylphosphate, SPCE: screen-printed carbon electrode, PEDOT/PSS: poly (3,4-ethylenedioxythiophene) polystyrene sulfonate.

## Data Availability

The data presented in this study are available on request from the corresponding author.
